# Low prevalence of lipid metabolism abnormalities in *APOE* ε2-genotype and male patients 60 years or older with schizophrenia

**DOI:** 10.1186/s12888-017-1530-9

**Published:** 2017-12-12

**Authors:** Chunxia Ban, Qunying Zhang, Jie Feng, Huijuan Li, Qi Qiu, Yuan Tian, Xia Li

**Affiliations:** 1 Department of Psychogeriatrics, Shanghai Mental Health Center, Shanghai Jiao Tong University School of Medicine, South Wanping Road 600, Shanghai, 200030 China; 2 Mental Health Center of Jiading District in Shanghai, Shanghai, 201800 China; 3 Mental Health Center of Fengxian District in Shanghai, Shanghai, 201400 China

**Keywords:** *APOE*, Elderly schizophrenia, Glucose, Cholesterol, LDL, HDL, Triglycerides

## Abstract

**Background:**

Schizophrenia is a serious mental disorder largely manageable with atypical antipsychotics; however, these drugs have been associated with glucose/lipid metabolism issues such as diabetes and hyperlipidaemia. Apolipoprotein E (*APOE*) is the most abundant apolipoprotein, and *APOE* genotypes have been correlated with lipid metabolism phenotypes in an age-dependent manner. Studies examining the relationship between the *APOE* genotype and lipid abnormalities in patients with schizophrenia have been inconclusive, but primarily focused on adult patient populations. Therefore, we explored the correlations between the *APOE* genotype and glucose/lipid metabolism indicators and abnormalities in hospitalized patients 60 years or older with schizophrenia with a history of long-term antipsychotics use.

**Methods:**

We assessed *APOE* genotype, age, weight, height, blood glucose, triglycerides, cholesterol, high-density lipoprotein, and low-density lipoprotein in a total of 294 patients. *APOE* genotypes were divided into three groups: *APOE* ε2 (ε2/ε2 and ε2/ε3), *APOE* ε3 (ε3/ε3), and *APOE* ε4 (ε3/ε4 and ε4/ε4), and comparisons were conducted among these groups or according to ε2 carrier status.

**Results:**

*APOE* ε3/ε3 was the most common genotype (68.3%) and at least one ε3 allele was present in 81.8% of patients. There were no differences in antipsychotics type or dose according to the *APOE* genotype, but serum cholesterol values varied near significantly (*P* = 0.052) and low-density lipoprotein values varied significantly according to genotype (*P* < 0.05, lowest in the *APOE* ε2 genotype). Men had lower cholesterol and low-density lipoprotein levels (*P* < 0.05) than women. Compared to patients administered typical antipsychotics, those administered atypical antipsychotics had higher triglyceride, cholesterol, and low-density lipoprotein levels (*P* < 0.05). Stepwise linear regressions showed that cholesterol and low-density lipoprotein levels were influenced by sex, the *APOE* ε2 genotype, and atypical antipsychotics use.

**Conclusions:**

In the context of atypical antipsychotics use, carriers of the *APOE* ε2-genotype and male patients with schizophrenia 60 years or older may be less likely to develop a lipid metabolism abnormality.

## Background

Schizophrenia is a severe mental illness that has a lifetime risk of about 1% [1]. The prognosis for schizophrenia is typically poor with a low recovery rate [2]. Schizophrenia imposes burdens on not only the affected individuals but also their families as well as society. Accordingly, it is a serious public health problem and social issue [3].

The main clinical manifestations of schizophrenia include positive symptoms, negative symptoms, and cognitive impairment [4, 5]. Atypical antipsychotics have good efficacy for these three symptom domains and produce fewer extrapyramidal reactions than typical antipsychotics. Yet, the ability of these drugs to cause weight gain, hyperglycaemia, and hyperlipidaemia in patients has garnered significant attention [6–8]. These side effects impact patient medication adherence and limit the treatment options available to physicians for patient management. Even so, not all patients who are administered atypical antipsychotics develop a glucose/lipid metabolic disorder. The reasons for this have yet to be clarified. It is important to explore and understand the risk factors and protective factors for schizophrenia treatment so that clinicians can make more informed and accurate treatment choices.

Apolipoprotein E (*APOE*) is the most abundant apolipoprotein [9, 10]. It has six genotypes (ε2/ε2, ε2/ε3, ε3/ε3, ε/ε4, ε3/ε4, ε4/ε4) originating from three different alleles (ε2, ε3, ε4) [11]. Some researchers have found that the *APOE* genotype is related to the manifestation of glucose/lipid metabolic abnormalities in patients without schizophrenia [12–14]. However, the relationships among *APOE* gene polymorphisms, blood sugar, and blood lipid content are not fully understood. This may be due to the age-dependent influence of *APOE* genotype on glucose/lipid metabolism [15, 16], as most previous studies do not account for the potential effects of age [13]. Most studies of patients with schizophrenia have been conducted with subjects who were mainly under the age of 60 years. These studies have explored the relationship between the *APOE* genotype and susceptibility to schizophrenia, but not that between the *APOE* genotype and metabolic syndrome [17, 18]. Elderly individuals are more likely to develop glucose/lipid metabolic abnormalities than their younger counterparts [19–21]. Thus, we conducted a study on Han Chinese elderly patients (60 years or older) with schizophrenia and long-term history of antipsychotics use. We evaluated the *APOE* genotype, blood sugar, blood lipids, and other related indicators to explore the influence of *APOE* polymorphisms on lipid and glucose metabolism in hospitalized patients 60 years or older with schizophrenia.

## Methods

### Study design and participants

**Fig. 1:**
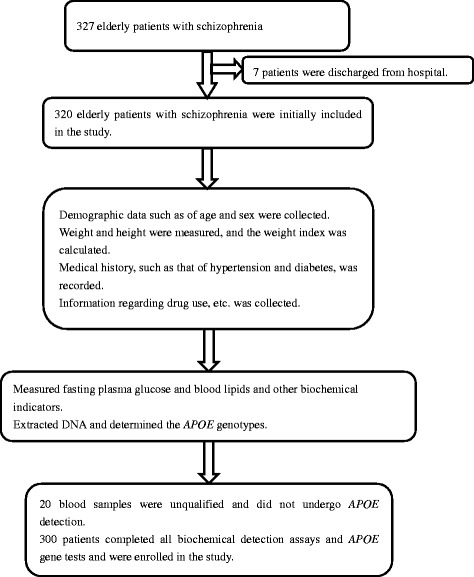
Flowchart depicting the selection of elderly patients with schizophrenia

The study was conducted at three mental health centres in Shanghai, China (the Shanghai Mental Health Center, the Mental Health Center of Jiading District in Shanghai, and the Mental Health Center of Fengxian District in Shanghai) between July 1, 2015 and December 31, 2015 (Fig. 1). Information regarding sex, age, height, weight, body mass index (BMI), and current prescribed medicines was recorded for all patients. Obesity was classified according to the health of the People’s Republic of China industry standard WS/T 428–2013, which defines overweight as a BMI ≥ 24.0. Schizophrenia was diagnosed by a senior psychiatrist according to the International Classification of Diseases 10 diagnostic standard. Diabetes was previously diagnosed by an endocrinologist according to the World Health Organization 1999 criteria [22]. High blood pressure was defined as high average measured blood pressure (systolic blood pressure ≥ 140 mmHg or diastolic blood pressure ≥ 90 mmHg) or previous diagnosis by a clinical specialist.

### Measurements of blood sugar and blood lipids

Research subjects fasted from 20:00 in the evening prior to blood sampling and were prohibited from drinking water for 4 h prior to blood sampling. A total of 5 mL of venous blood was collected from each patient in the fasting state between 06:30 and 08:30. Samples were incubated at room temperature for 30 min and then centrifuged at 3000 rpm for 15 min to collect the serum. All samples were tested for serum glucose, triglyceride content, cholesterol content, high-density lipoprotein, and low-density lipoprotein.

### *APOE* genotyping by polymerase chain reaction (PCR)-ligase detection reaction (LDR)

A total of 5 mL of peripheral blood was slowly injected into an EDTA-coated anticoagulant tube, shaken, and then centrifuged at 3000 rpm for 20 min at 4 °C. DNA in 1 mL of blood was extracted with a whole blood genome DNA isolation kit (spin column, Tiangen Biochemical Science and Technology Co., Ltd., Beijing, China).

Multiplex PCR reactions on the basis of the two SNP cores of the *APOE* gene were conducted using the rs429358 and rs7412 design primers as follows: P1:5′-GCCTACAAATCGGAACTGGA CAGCTCCTCGGTGCTCTG-3′ and P2:5′-TAAGCGGCTCCTCCGCGATGCCCCGGCCTGGTACACTG-3′. The total reaction volume was 20 μL and included the following: 1 μL (50 ng) genomic DNA, 2 μL 1e reaction buffer, 0.6 μL 3 mM Mg^2+^, 2 μL of 2 mM of each dNTP, 0.2 μL 1 U Taq enzyme, 12.2 μL double-distilled H_2_O (ddH_2_O), and 2 μL 0.5p Primer Mix. PCR products were examined using 3.0% agarose gel electrophoresis.

Details of the multiplex LDR reaction can be found in Table 1. The reaction volume was 10 μL and included the following: 1 μL reaction buffer, 1 μL 2 pmol/μL Probe Mix (each), 0.05 μL 2 U Taq DNA ligase, 4 μL ddH_2_O, and 4 μL PCR product. Finally, *APOE* genotypes were assigned according to product fragment sizes on 3.0% agarose gel electrophoresis (Table 2). All genotyping was performed in duplicate.

**Table 1:** Reaction probes used in the multiplex ligase detection reaction

PROBE NAME	SEQUENCE (5′-3′)	LDR length
rs429358_modify	P-CACGTCCTCCATGTCCGCGCTTTTTTTTTTTTTTTTTTTTTTTTTTT-FAM	
rs429358_C	TTTTTTTTTTTTTTTTTTTTTTCGGTACTGCACCAGGCGGCCGCG	92
rs429358_T	TTTTTTTTTTTTTTTTTTTTTTTTCGGTACTGCACCAGGCGGCCGCA	94
rs7412_modify	P-CTTCTGCAGGTCATCGGCATTTTTTTTTTTTTTTTTTTTTTTTTTTTTTT-FAM	
rs7412_C	TTTTTTTTTTTTTTTTTTTTTTTTCCGGCCTGGTACACTGCCAGGCG	97
rs7412_T	TTTTTTTTTTTTTTTTTTTTTTTTTTCCGGCCTGGTACACTGCCAGGCA	99

Note: *LDR* ligase detection reaction

**Table 2:** *APOE* genotype determination method

*APOE* genotype	Rs429358	Rs7412
ε2/ε2	TT	TT
ε2/ε3	TT	TC
ε3/ε3	TT	CC
ε2/ε4	TC	TC
ε3/ε4	TC	CC
ε4/ε4	CC	CC

### Data analysis

All data were inputted using the EpiData3.1 software and analysed with the SPSS 17.0 software package (SPSS Inc., Chicago, IL). *APOE* genotypes were calculated by alignment inspection according to the Hardy–Weinberg law. Continuous data (age, chlorpromazine equivalent dose, triglycerides, cholesterol, high-density lipoprotein, low-density lipoprotein, and fasting plasma glucose) are represented with descriptive statistics as means ± standard deviation. Categorical data (sex, overweight status, diabetes, high blood pressure, atypical antipsychotics use, lipid-lowering medication use, and glucose-lowering medication use) are represented as percentages of the total population. *APOE* genotypes were divided according to three categories: *APOE* ε2 (ε2/ε2 and ε2/ε3), *APOE* ε3 (ε3/ε3), and *APOE* ε4 (ε3/ε4 and ε4/ε4). Patients with the ε2/ε4 genotype were excluded from analysis because of known opposite effects of ε2 and ε4 on lipid levels. Inpatients who were ε2 carriers or non-ε2 carriers were labelled as *APOE* ε2+ or *APOE* ε2-, respectively. Continuous data were analysed between two groups using independent sample *t* tests and among three groups using a one-way analysis of variance. Categorical data were analysed using chi-squared (*χ* ^*2*^) tests; however, atypical antipsychotics use and lipid-lowering medication use were analysed using Fisher’s exact test. Low-density lipoprotein was examined with pairwise comparisons using Hochberg’s GT2(H) method, while cholesterol was examined with pairwise comparisons using the Games-Howell(A) method. Finally, a regression analysis was performed on cholesterol, low-density lipoprotein, and potential influencing factors. The significance level was set at *P* < 0.05.

## Results

### Demographic characteristics and *APOE* genotype distribution

**Fig. 2:**
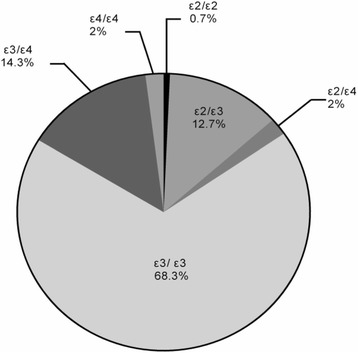
*APOE* genotype distribution in elderly patients with schizophrenia

All patients were of Han Chinese descent. There were 160 men (53.3%) and 140 women (46.7%). The mean age was 67.3 ± 6.66 years (range, 60–92 years). Two of the participants had the ε2/ε2 *APOE* genotype, 38 participants were ε2/ε3, 6 were ε2/ε4, 205 participants (were ε3/ε3, 43 were ε3/ε4, and 6 were ε4/ε4; Fig. 2). According to the Hardy-Weinberg law, there was *χ* ^*2*^ = 243.79, *df* = 3, and *P* > 0.05. This indicates that the distribution of the genotype was in accordance with Hardy-Weinberg equilibrium. The allele frequencies were as follows: ε2 = 48 (8.0%), ε3 = 491 (81.8%), and ε4 = 61 (10.2%).

### Comparison of clinical characteristics among the *APOE* genotype groups

Statistically significant differences were observed for lipid-lowering medication use and low-density lipoprotein according to the *APOE* genotype (*P* < 0.05). Near statistically significant differences were observed for cholesterol according to the *APOE* genotype (*P* = 0.052). There were no statistically significant differences in sex, age, high blood pressure, diabetes, glucose-lowering medication use, atypical antipsychotics use, plasma glucose, triglycerides, or high-density lipoprotein according to genotype (*P* > 0.05) (Table 3).

**Table 3:** Comparison of clinical characteristics according to *APOE* genotype in elderly patients with schizophrenia

Item		*APOE* ε2(*n* = 40)	*APOE* ε3(*n* = 205)	*APOE* ε4(*n* = 49)	*x* ^*2*^ */F*	*P-value*
Men (%)		23 (57.5%)	109 (53.2%)	26 (53.1%)	0.263	0.877
Age (years)		67.65 ± 7.17	67.10 ± 6.54	68.00 ± 6.58	0.422	0.656
Overweight status (%)		18 (45.0%)	98 (47.8%)	21 (42.9%)	0.437	0.804
History of disease	Diabetes (%)	11 (27.5%)	52 (25.4%)	12 (24.5%)	0.112	0.945
	High blood pressure (%)	11 (27.5%)	78 (38.0%)	19 (38.8%)	1.708	0.426
Drug use*	Atypical antipsychotic use (%)	36 (90.0%)	179 (87.3%)	45 (91.8%)		0.751#
	Chlorpromazine equivalent dose (mg)	322.88 ± 320.06	318.52 ± 254.79	361.98 ± 371.20	0.459	0.490
	Lipid-lowering mediation use (%)	1 (2.5%)	6 (2.9%)	7 (14.3%)		0.006#
	Glucose-lowering medication use (%)	9 (22.5%)	43 (21.0%)	12 (24.5%)	0.296	0.863
Serum biochemical indices&	Triglycerides (mmol/L)	1.35 ± 0.81	1.38 ± 0.84	1.43 ± 0.80	0.062	0.940
	Cholesterol (mmol/L)	4.38 ± 0.91	4.74 ± 0.91	4.94 ± 1.47	2.994	0.052
	High-density lipoprotein (mmol/L)	1.36 ± 0.35	1.29 ± 0.41	1.25 ± 0.45	0.812	0.445
	Low-density lipoprotein (mmol/L)	2.34 ± 0.63	2.83 ± 0.75	2.85 ± 0.98	7.038	0.001
	Plasma glucose (mmol/L)	5.58 ± 1.40	5.50 ± 1.49	5.27 ± 0.89	1.198	0.303

Note: *APOE* genotypes were divided into three groups: an *APOE* ε2 group (ε2/ε2 and ε2/ε3), an *APOE* ε3 group (ε3/ε3), and an *APOE* ε4 group (ε3/ε4 and ε4/ε4)

#: Fisher’s Exact Test

*: Different antipsychotics were converted to chlorpromazine equivalent doses

&: Plasma glucose was compared among the three groups with glucose-lowering medication use as a covariate. Serum lipids were compared among the three groups with lipid-lowering medication use as a covariate

### Comparisons of blood sugar, blood lipids, and other related indicators according to *APOE* genotype, ε2 allele carrier status, sex, and drug use

**Fig. 3:**
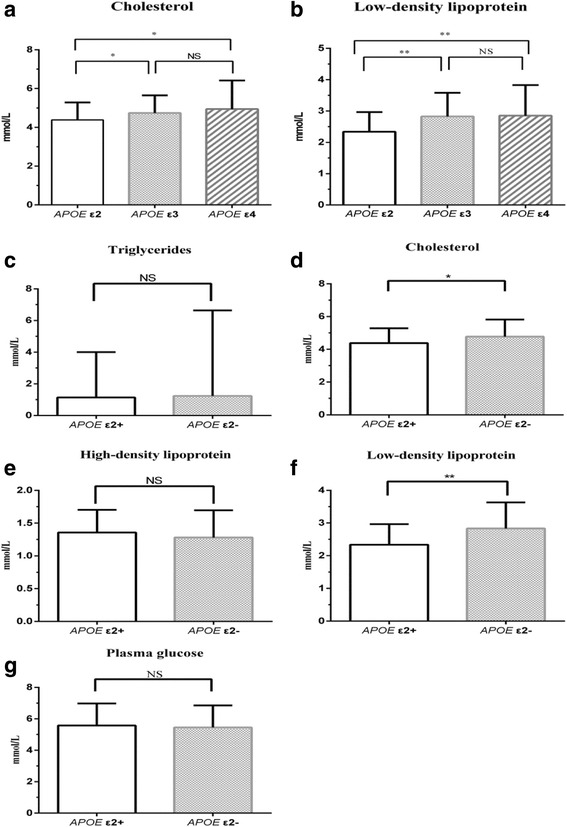
Comparisons of blood sugar, blood lipids, and other related indicators according to *APOE* genotype, (**a**-**b**), ε2 allele carrier status (**c**-**g**) in elderly patients with schizophrenia. Note: *APOE* ε2+ indicates carrier status for the ε2 allele and *APOE* ε2- indicates non-carrier status for the ε2 allele. * indicates *P* < 0.05, ** indicates *P* < 0.01, and NS indicates non-significant, *P* > 0.05

With lipid-lowering medication used as a covariate, cholesterol was significantly lower in elderly patients with schizophrenia with the *APOE* ε2 genotype than in those with the *APOE* ε3 and *APOE* ε4 genotypes (*P* < 0.05). No statistically significant differences were identified between the *APOE* ε3 and *APOE* ε4 genotypes (*P* > 0.05) (Fig. 3a). Low-density lipoprotein was also lower in patients with the *APOE* ε2 genotype than in those with the *APOE* ε3 and *APOE* ε4 genotypes (*P* < 0.01). No statistically significant differences were identified between the *APOE* ε3 and *APOE* ε4 genotypes (*P* > 0.05) (Fig. 3b). Cholesterol and low-density lipoprotein were lower in ε2 allele carriers with *APOE* ε2+ genotypes than in those with *APOE* ε2- genotypes (independent sample *t* tests, *P* < 0.05). No statistically significant differences were identified for triglycerides, plasma glucose, or high-density lipoprotein in ε2 allele carriers (*P* > 0.05) (Figs. 3c–g).

Women had higher cholesterol levels (5.02 ± 1.10 vs. 4.47 ± 0.90 nmol/L, *P* < 0.01) and higher low-density lipoprotein levels (2.88 ± 0.81 vs. 2.66 ± 0.77 nmol/L, *P* < 0.05) than men. Compared to patients administered typical antipsychotics, those administered atypical antipsychotics had higher triglyceride levels (1.43 ± 0.86 vs. 1.09 ± 0.43 nmol/L, *P* < 0.01), higher cholesterol levels (4.78 ± 1.05 vs. 4.33 ± 0.85 nmol/L, *P* < 0.05), and higher low-density lipoprotein levels (2.80 ± 0.80 vs. 2.46 ± 0.72 nmol/L, *P* < 0.05).

### Regression analysis of abnormal cholesterol and low-density lipoprotein in elderly patients with schizophrenia

Stepwise linear regressions were conducted with cholesterol, low density lipoprotein as the dependent variable and age, sex (0 = women, 1 = men), overweight status (0 = no, 1 = yes), *APOE* ε2 genotype (0 = no, 1 = yes), *APOE* ε3 genotype (0 = no, 1 = yes), *APOE* ε4 genotype (0 = no, 1 = yes), atypical antipsychotic drug use (0 = no, 1 = yes), diabetes (0 = no, 1 = yes), high blood pressure (0 = no, 1 = yes), and lipid-lowering medication use (0 = no, 1 = yes) as independent variables. The results showed that cholesterol and low-density lipoprotein were influenced by sex, *APOE* ε2 genotype, and atypical antipsychotics use (Table 4).

**Table 4:** Regression analysis of abnormal cholesterol and low-density lipoprotein in elderly patients with schizophrenia

Variables		Regression coefficients	Standardized regression coefficients	*t-value*	*P-value*
Dependent variable	Independent variables				
Total cholesterol^a^	Male sex	−0.533	−2.058	−4.626	0.000
	APOEε2+	−0.382	−0.127	−2.279	0.023
	Atypical antipsychotic use	0.409	0.127	2.273	0.024
Low-density lipoprotein^b^	Male sex	−0.200	−0.126	−2.227	0.027
	APOEε2+	−0.493	−0.213	−3.783	0.000
	Atypical antipsychotic use	0.336	0.136	2.404	0.017

Note: ^a^Coefficient of determination *R* ^***2***^ **=** 0.104, *F* = 11.181; ^b^Coefficient of Determination *R* ^*2*^ **=** 0.082,*F* = 8.631

## Discussion

Davignon et al. [23] found that *APOE* polymorphisms are important genetic factors influencing blood lipids, such as cholesterol. One study found that the presence of the *APOE* ε4 allele was significantly correlated with high serum cholesterol and low-density lipoprotein. ε2 and ε4 have opposite effects on lipid levels. ε4 allele carriers had the highest cholesterol and low-density lipoprotein values, followed by carriers of the ε3 allele, with ε2 allele carriers having the lowest values [24]. It has also been suggested that the presence of the ε2 allele reduces low-density lipoprotein [25] and that the ε4 allele increases low-density lipoprotein [26]. These findings are in agreement with our results in elderly patients with schizophrenia; serum cholesterol and low-density lipoprotein values were significantly lower in patients with the ε2 allele, while they were significantly higher in patients with the ε4 allele. Taken together, these data show that the effects of the *APOE* genotype on serum blood lipids are consistent between patients with schizophrenia and healthy subjects.

It is worth noting that the elderly patients with schizophrenia in this study had specifically been administered atypical antipsychotic drugs for years. In this context, ε2 carriers had more healthy cholesterol and low-density lipoprotein values than ε3 or ε4 carriers. These data suggest that ε2 carrier status is a protective factor against lipid metabolism abnormalities. This hypothesis was corroborated by a regression analysis. Therefore, we deduced that even after being administered atypical antipsychotics for a long time, elderly *APOE* ε2 carriers with schizophrenia may have a lower occurrence of lipid metabolism abnormalities than ε2 non-carriers. This is reflected in the fact that the ε2 carriers had the lowest rate of lipid-lowering drug consumption. Further investigations are required to explore whether the ε2 genotype can protect patients from metabolic problems during the initial years of antipsychotics treatment.

Studies have reported sex differences in the clinical features of schizophrenia [27]. Moreover, it has been reported that antipsychotic drugs exert sex-specific influences on blood lipids (i.e., larger effects on women). Consistent with this research, we found that female elderly patients with schizophrenia had higher serum lipid levels and were more likely to have lipid metabolism abnormalities than men.

At present, antipsychotic drugs are the first-line therapy for the treatment of schizophrenia. Among them, atypical antipsychotics are common but can cause weight gain as well as greatly increase a patient’s risk of developing diabetes and other metabolic syndromes [28, 29]. Moreover, the wide application of atypical antipsychotics has revealed that these drugs have a larger influence on blood lipids than typical antipsychotics [30–32]. In the present study, we found similar results and confirmed this effect of atypical antipsychotics use.

The correlation between the *APOE* genotype and triglycerides has been inconsistently reported in previous studies [23, 33]. A study with elderly Brazilian women found that triglyceride values were higher in ε2 carriers than in ε4 carriers [34]. In contrast, no obvious relationship between *APOE* status and serum triglycerides was reported in a study with patients with metabolic syndrome [35], which is in line with our findings. Moreover, the lack of correlation between high density lipoprotein and the *APOE* genotype that we noted in this study is consistent with other results [36].

In a previous study with a community sample of elderly Han Chinese individuals, we showed a potential association between the *APOE* ε3/ε3 genotype and increased susceptibility to diabetes [13]. However, this study showed that there were no statistical differences among the three genotype groups. We consider that these results require confirmation in a larger community sample, as the use of a hospitalized sample in the present study may have influenced our results.

A major factor limiting the generalizability of this study was our selection of hospitalized elderly patients with schizophrenia; specifically, the inclusion of long-term hospitalized patients with a relatively fixed diet and regular blood glucose monitoring. The influence of hospital environmental factors on glucose/lipid metabolism cannot be completely discounted, and accordingly our results should be validated in a community sample of elderly patients with schizophrenia. Moreover, we did not include patients who were abstinent from antipsychotic medication or non-elderly patients as control subjects. Therefore, this study does not inform us regarding the effects of specific antipsychotic drugs or those of age on glucose/lipid metabolism. In addition, the study results may be subject to survival bias. We thus could not assess the effects of the *APOE* genotype on the lifespan of elderly patients. Future studies should address these limitations.

## Conclusion

Our study showed that the *APOE* ε2 allele and male sex are possible protective factors against blood lipid abnormalities in the context of atypical antipsychotic use in patients with schizophrenia 60 years or older. This finding informs the relationship between *APOE* genotype status and glucose/lipid metabolism in patients with schizophrenia of Han ethnicity (and possibly other Asian ethnicities), and can assist the development of a more personalized approach to schizophrenia treatment selection.

## Abbreviations

*APOE*: Apolipoprotein E
BMI: Body mass index
LDR: Ligase detection reaction

## References

[1] Purcell SM, Wray NR, Stone JL, Visscher PM, O'Donovan MC, Sullivan PF (2009). Common polygenic variation contributes to risk of schizophrenia and bipolar disorder. Nature.

[2] Jobe TH, Harrow M (2010). Schizophrenia course, long-term outcome, recovery, and prognosis. Curr Dir Psychol Sci.

[3] Long J, Huang G, Liang W, Liang B, Chen Q, Xie J (2014). The prevalence of schizophrenia in mainland China: evidence from epidemiological surveys. Acta Psychiatr Scand.

[4] Rezaee O, Saeede MM, Reza M, Akbarpour FA (2012). Placebo-controlled trial of bupropion for improving the positive and negative symptoms of schizophrenia. International Journal of Collaborative Research on Internal Medicine & Public Health.

[5] Soštarič M, Zalar B (2011). The overlap of cognitive impairment in depression and schizophrenia: a comparative study. Psychiatr Danub.

[6] Sernyak MJ, Leslie DL, Alarcon RD, Losonczy MF, Rosenheck R (2002). Association of diabetes mellitus with use of atypical neuroleptics in the treatment of schizophrenia. Am J Psychiatry.

[7] Tajima K, Fernández H, López-Ibor JL, Carrasco JL, Díaz-Marsá M (2009). Schizophrenia treatment. Critical review on the drugs and mechanisms of action of antipsychotics. Actas Espanolas De Psiquiatria.

[8] Cüneyt Ü, Yakup A, Neslihan A, Murat K, Kenji H (2013). Reduced serum paraoxonase 1 (PON1) activity in patients with schizophrenia treated with olanzapine but not quetiapine. Neuropsychiatr Dis Treat.

[9] Hu J, Liu CC, Chen XF, Zhang YW, Xu H, Opposing BG (2015). Effects of viral mediated brain expression of apolipoprotein E2 (apoE2) and apoE4 on apoE lipidation and Aβ metabolism in apoE4-targeted replacement mice. Mol Neurodegener.

[10] Feo ED, Simone B, Persiani R, Cananzi F, Biondi A, Arzani D (2012). A case–control study on the effect of Apolipoprotein E genotypes on gastric cancer risk and progression. BMC Cancer.

[11] Al-Dabbagh NM, Al-Dohayan N, Arfin M, Tariq M, Apolipoprotein E (2009). Polymorphisms and primary glaucoma in Saudis. Mol Vis.

[12] Chaudhary R, Likidlilid A, Peerapatdit T, Tresukosol D, Srisuma S, Ratanamaneechat S (2012). Apolipoprotein E gene polymorphism: effects on plasma lipids and risk of type 2 diabetes and coronary artery disease. Cardiovasc Diabetol.

[13] Ban C, Zhong L, Wang T, Zhu M, Wang J, Zhang Z (2016). Enhanced diabetes susceptibility in community dwelling Han elders carrying the Apolipoprotein E 3/3 genotype. PLoS One.

[14] Lin SK, Kao JT, Tsai SM, Tsai LY, Lin MN, Lai CJ (2004). Association of apolipoprotein E genotypes with serum lipid profiles in a healthy population of Taiwan. Ann Clin Lab Sci.

[15] Scuteri A, Najjar SS, Muller D, Andres R, Morrell CH, Zonderman AB (2005). apoE4 allele and the natural history of cardiovascular risk factors. Am J Physiol Endocrinol Metab.

[16] Igbavboa U, Eckert GP, Malo TM, Studniski AE, Johnson LN, Yamamoto N (2005). Murine synaptosomal lipid raft protein and lipid composition are altered by expression of human apoE 3 and 4 and by increasing age. J Neurol Sci.

[17] González-Castro TB, Fresán A, Juárez-Rojop IE, Ble-Castillo JL, López-Narváez L, Genis A (2015). No association between ApoE and schizophrenia: evidence of systematic review and updated meta-analysis. Schizophr Res.

[18] Saeed Mohammad AA, Saeed K, Misbahul A, Mohammad T, Abdulrahman AA (2015). Apolipoprotein E polymorphism is associated with susceptibility to schizophrenia among Saudis. Archives of Medical Science Ams.

[19] Chen GY, Li L, Dai F, Li XJ, XX X, Fan JG (2015). Prevalence of and risk factors for type 2 diabetes mellitus in hyperlipidemia in China. Medical science monitor international medical journal of experimental & Clinical Research.

[20] Longombenza B, On'Kin JB, Okwe AN, Kabangu NK, Fuele SM (2010). Metabolic syndrome, aging, physical inactivity, and incidence of type 2 diabetes in general African population. Diabetes & Vascular Disease Research Official Journal of the International Society of Diabetes & Vascular Disease.

[21] Carniciu S, Caceaune E, Mihai A, Zetu C, Ionescutîrgovişte C (2013). The prevalence of overweight and obesity in newly discovered diabetic patients. Romanian journal of Diabetes Nutrition & Metabolic Diseases.

[22] Alberti KGMM, Zimmet PZ (1998). Definition, diagnosis and classification of diabetes mellitus and its complications. Part 1: diagnosis and classification of diabetes mellitus. Provisional report of a WHO consultation. Diabet Med.

[23] Davignon J, Bouthillier D, Nestruck AC, Sing CF, Apolipoprotein E (1988). Polymorphism and atherosclerosis: insight from a study in octogenarians. Arteriosclerosis.

[24] Hagberg JM, Wilund KR, Ferrell REAPOE (2000). gene and gene-environment effects on plasma lipoprotein-lipid levels. Physiol Genomics.

[25] Mazzotti DR, Singulane CC, Ota VK, Rodrigues TP, Furuya TK, de Souza FJ (2014). Association of APOE, GCPII and MMP9 polymorphisms with common diseases and lipid levels in an older adult/elderly cohort. Gene.

[26] Borilova LP, Bartova J, Poskerova H, Machal J, Vokurka J, Fassmann A (2015). Apolipoprotein E gene polymorphisms in relation to chronic periodontitis, periodontopathic bacteria, and lipid levels. Arch Oral Biol.

[27] Zhang XY, Chen DC, Xiu MH, Yang FD, Haile CN, Kosten TA (2012). Gender differences in never-medicated first-episode schizophrenia and medicated chronic schizophrenia patients. J. Clin. Psychiatry..

[28] Wysokiński A, Kowman M, Kłoszewska I (2012). The prevalence of metabolic syndrome and Framingham cardiovascular risk scores in adult inpatients taking antipsychotics - a retrospective medical records review. Psychiatr Danub.

[29] Kagal UA, Torgal SS, Patil NM, Malleshappa A (2012). Prevalence of the metabolic syndrome in schizophrenic patients receiving second-generation antipsychotic agents--a cross-sectional study. J Pharm Pract.

[30] Aquila R, Emanuel M (2000). Interventions for weight gain in adults treated with novel antipsychotics. Prim. Care companion. J. Clin. Psychiatry..

[31] Rc S, Jp L, N B, J W, S V, A K. Clozapine, risperidone, olanzapine, and conventional antipsychotic drug effects on glucose, lipids, and leptin in schizophrenic patients. Int J Neuropsychopharmacol 2005;8:183–194. 10.1017/S1461145705005110 15737248

[32] Bergman RN, Ader M (2005). Atypical antipsychotics and glucose homeostasis. J Clin Psychiatry.

[33] Sofat R, Cooper JA, Kumari M, Casas JP, Mitchell JP, Acharya J (2016). Circulating Apolipoprotein E concentration and cardiovascular disease risk: meta-analysis of results from three studies. PLoS Med.

[34] Paula RS, Souza VC, Benedet AL, Souza ER, Toledo JO, Moraes CF (2010). Dietary fat and apolipoprotein genotypes modulate plasma lipoprotein levels in Brazilian elderly women. Mol Cell Biochem.

[35] Sun YP, Wei R, Yan DD, FL X, Zhang XJ, Zhang B (2016). Association between APOE polymorphism and metabolic syndrome in Uyghur ethnic men. BMJ Open.

[36] Eggertsen G, Tegelman R, Ericsson S, Angelin B, Berglund L, Apolipoprotein E (1993). Polymorphism in a healthy Swedish population: variation of allele frequency with age and relation to serum lipid concentrations. Clin Chem.

